# HLA-vaccine transplantations for refractory leukemia: beyond double-unit cord blood

**DOI:** 10.3389/fimmu.2025.1726216

**Published:** 2025-12-05

**Authors:** Wing Leung, Michaela Su-Fern Seng, Thi Ngoc Anh Pham, Debbra Chong, Ah Moy Tan

**Affiliations:** 1 Children’s Blood and Cancer Centre, KK Women’s and Children’s Hospital, SingHealth, Singapore, Singapore; 2 Haematology/Oncology Service, Department of Paediatric Subspecialties, KK Women’s and Children’s Hospital, SingHealth, Singapore, Singapore; 3 Duke-National University of Singapore Medical School, Singapore, Singapore; 4 Nursing Clinical Services, Division of Nursing, KK Women’s and Children’s Hospital, SingHealth, Singapore, Singapore

**Keywords:** HLA, leukemia, allogeneic stem cell transplantation, graft verses leukemia response, alternative donor sources

## Abstract

Recent studies in double-unit cord blood transplantation have shown that when the patient (PT) and the losing unit (LU) share an HLA determinant mismatched against the winning unit (WU), leukemia relapse risk declines without added graft-versus-host disease (GvHD), suggesting a potential “HLA-vaccine” effect. Building on this principle, we treated five patients with refractory leukemia using graft combinations beyond cord–cord pairs, including haploidentical–cord (haplo-cord) and haploidentical–haploidentical (haplo-haplo) transplants, purposefully selected for predicted HLA-vaccine effects. All patients had measurable residual disease at transplantation and received reduced-intensity conditioning without total body irradiation. Engraftment occurred in all cases, with durable WU dominance and complete remission achieved within one month. Transient chimerism of the LU suggests a rapid, immune-mediated graft-versus-graft (GvG) and potentially graft-versus-leukemia (GvL) effect, triggered by shared, mismatched HLA allotypes. These findings imply that HLA-vaccine transplantation may be feasible across diverse graft pairings, offering a novel therapeutic avenue for refractory leukemia.

## Introduction

In double-unit cord blood transplantations, recent studies have demonstrated that if the patient (PT) and the losing unit (LU) share the same HLA determinant mismatched against the winning unit (WU), the risk of leukemia relapse decreases without an increase in graft-versus-host disease (GVHD) (1, 2). These observations support the hypothesis that the mismatched antigen in the LU can serve as a “vaccine” for the WU against the PT’s leukemia cells (2). In practice, such favorable pairs of cord blood units may not always be available for all candidates of hematopoietic stem cell transplantation (HSCT). We herein report 5 patients with refractory leukemia who received other combinations of double-unit transplantations, such as haploidentical–cord blood (haplo-cord) or haploidentical–haploidentical (haplo-haplo) HSCT. Their donor pairs were selected purposefully for the HLA-vaccine effect, irrespective of which graft ultimately predominated.

## Patients and methods

All 5 patients had poor-prognosis leukemia with persistent measurable residual disease (MRD) at the time of vaccine transplantation (**Table 1**).

**Table 1:** Patient characteristics and outcomes.

Age	Primary disease	Prior HSCT	Prior CAR-T/antibody	Disease status at HSCT referral	Bridging therapy before vaccine HSCT	Disease status at vaccine HSCT	Vaccine graft – winning graft	Vaccine antigen (HLA)	Conditioning	Acute GVHD	Chronic GVHD	Status	Time after vaccine HSCT
7	T-ALL TCF7:: SPI1	No	No	Day 33 MRD 1.61%	Protocol 1b HDMTX Nelarabine Dasatinib	BM MRD 5x10-5	Haplo – UCB	A, DRB1	Flu-Bu-TT-Cy-AraC	None	None	Alive in CR, MRD–	9m
12	T-ALL NUP98:: RAP1-GDS1	No	No	Day 37 BM Blasts 46.4%	Protocol 1b Nelarabine Venetoclax	BM MRD 1x10-5	MSD – Haplo	A, B, C, DRB1	Flu-Bu-TT-Cy-AraC	Grade 3, Skin, Gut	Moderate, Lung	Alive in CR, MRD–	9m
15	B-ALL MEF2D:: BCL9	1.Haplo 2.Haplo	1.Tisa-Cel 2.Tisa-Cel 3.CD19/22 /72 CAR-T	6^th^ relapse, Orbit	Radiation Venetoclax Sunitinib Bortezomib	BM MRD 5x10-5	UCB – Haplo	B, C, DRB1	Flu-Treo-TT-Cy-AraC	None	Moderate Skin, Eye Mouth, Liver	Alive in CR, MRD–	5m
6	B-ALL Hyper-diploid	1.MUD	1.Blinatu-momab 2.Inotuzu-mab	2^nd^ relapse, BM Blasts 78%	HDMTX Venetoclax Mini-hyper CVD	BM MRD 2.2%	UCB – Haplo	A	Flu-Bu-TT-Cy-AraC	None	None	Alive in CR, MRD–	5m
16	AML FLT3-ITD	1.MSD	No	1^st^ relapse, BM blasts 91%	Gilteritinib, Venetoclax Azacytidine	PB Blasts 54%	Haplo – Haplo	A, B, DRB1	Flu-Treo-TT-Cy-AraC	Grade 3, Gut Liver	None	Alive in CR, MRD–	3m

*MUD, matched unrelated donor; MSD, matched sibling donor; HDMTX, high-dose methotrexate; MRD, measurable residual disease; BM, bone marrow; PB, peripheral blood; Haplo, haploidentical; UCB, umbilical cord blood; Flu, fludarabine (160-200mg/m2); Bu, busulfan (10mg/kg); Treo, treosulfan (30mg/kg); TT, thiotepa (10mg/kg); Cy, cyclophosphamide (130-180mg/kg); AraC, cytarabine (200-900mg/m2); CR, complete remission.

**Figure 1:**
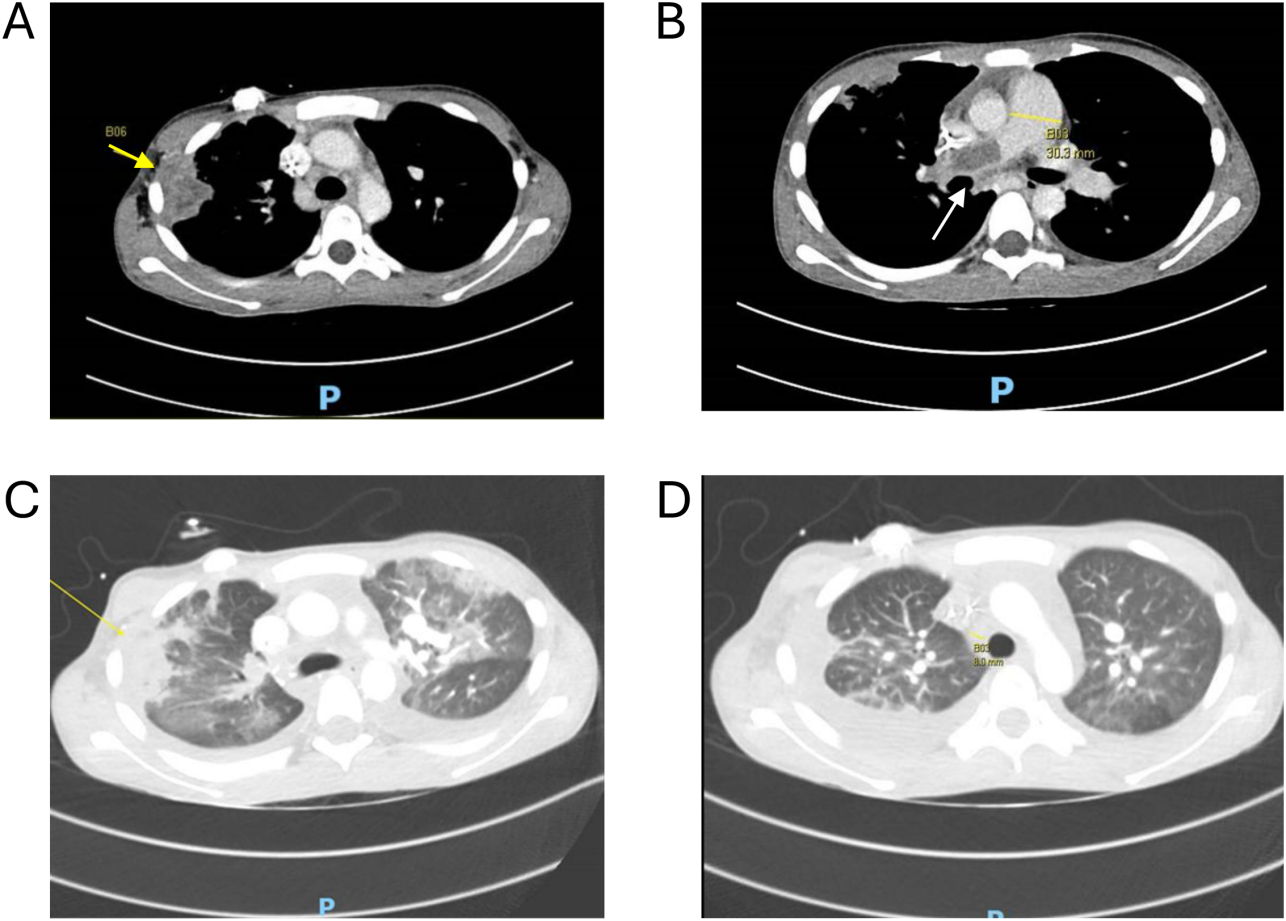
Angioinvasive pulmonary mucormycosis. **(A)** Peripheral consolidation with pleural and chest wall invasion (yellow arrow). **(B)** Extensive pulmonary embolus (white arrow) expanding the right main pulmonary artery (30.3mm) extending into the right lobar, segmental and subsegmental pulmonary arteries. **(C)** Extensive ground glass opacities scattered throughout both lungs. **(D)** Interval resolution one month after vaccine transplantation.

- Patient 1 had T-lineage acute lymphoblastic leukemia (T-ALL) with *TCF7::SPI1* gene fusion, previously associated with uniformly poor overall survival (3). As expected, she had high-level MRD (1.61%) post-induction and persistent disease despite intensive consolidation chemotherapy, including nelarabine and dasatinib.
- Patient 2 had T-ALL with *NUP98::RAP1GDS1* fusion, also linked to poor treatment response and short survival (4). She experienced induction failure with >40% blasts in the bone marrow on Day 37 of induction chemotherapy. After intensive salvage, including nelarabine and venetoclax, she still had persistent MRD.
- Patient 3 had B-ALL with high-risk *MEF2D::BCL9* fusion (5). His leukemia relapsed 6 times despite multimodality treatment, including two haploidentical HSCTs, two Tisa-Cel infusions, and one trial of triple-targeting αCD19/CD22/CD72 CAR T cells. At his 6th relapse, he had orbital extramedullary disease and MRD in the marrow, treated with radiation and multiagent therapy including venetoclax, sunitinib, and bortezomib.
- Patient 4 had hyperdiploid B-ALL. After his first marrow relapse, he had 43.3% residual blasts post-reinduction. Following 2 cycles of blinatumomab and TBI-containing (12 Gy) matched unrelated donor HSCT, he relapsed again 7 months later. Inotuzumab failed, resulting in a triple-negative (CD19–/CD20–/CD22–) phenotype. After bridging therapy with high-dose methotrexate, venetoclax, and mini–hyper-CVD regimen A, he had 2.27% residual blasts at the time of vaccine transplant.
- Patient 5 had AML with *FLT3-ITD*, relapsing within 4 months after consolidative HSCT (busulfan–cyclophosphamide conditioning, matched sibling donor). The AML was refractory to azacitidine/venetoclax reinduction and complicated by angio-invasive pulmonary mucormycosis (*Rhizopus*) involving the right main pulmonary artery, left lower lobar artery, right chest wall, and both lung extensively (**Figure 1**). After referral, mucormycosis was treated with thrombectomies, isavuconazole, and ambisome, while leukemia was treated with gilteritinib, venetoclax, and azacitidine. At vaccine transplant, he still had extensive pulmonary disease and ~50% circulating blasts.

### HLA vaccine transplant

All patients received non-TBI conditioning with fludarabine–busulfan/treosulfan–thiotepa–cyclophosphamide–cytarabine (6), with busulfan replaced by treosulfan if previously used. Doses were reduced-intensity (total busulfan 10 mg/kg, treosulfan 30 mg/kg, thiotepa 10 mg/kg, cytarabine 200–900 mg/m²), sufficient for engraftment but not for leukemia ablation, to limit morbidity in chemo-refractory disease. Tacrolimus and mycophenolate mofetil were given for GVHD prophylaxis.

## Results

**Figure 2:**
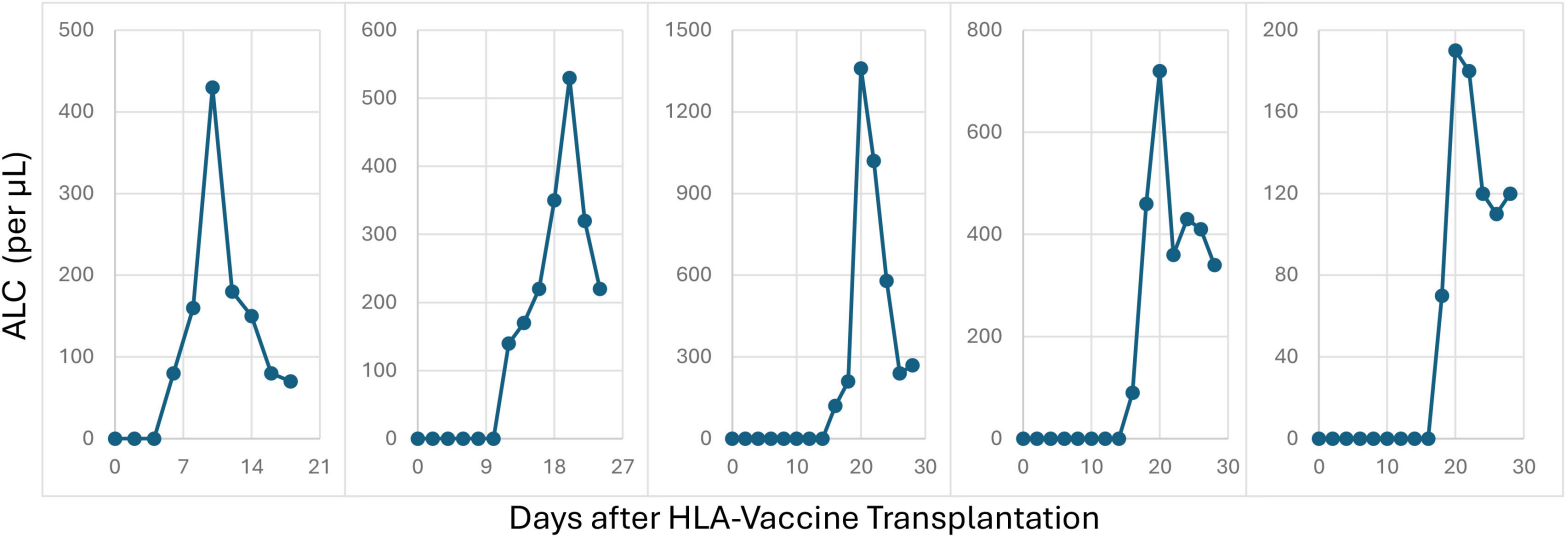
Absolute lymphocyte count. Absolute lymphocyte count (ALC) of the 5 patients peri-engraftment, demonstrating a pattern of transient surge in cell number followed by contraction.

The median number of HLA-vaccine antigens was 3 (range 1–4), involving both MHC Class I and II (**Table 1**). Median neutrophil engraftment occurred at 20 days (range 15–36). All patients had transient LU chimerism (median 17%, range 1–34%, peak Day 13) in peripheral blood, with WU subsequently reaching >90% chimerism at a median of 18 days (range 8–27). Around this period, a transient surge in lymphocyte count was seen in all patients (**Figure 2**). At last follow-up 3–9 months post-transplant, all patients maintained 100% WU-derived chimerism.

Two patients developed acute GVHD (both grade 3) and two developed chronic GVHD (both moderate), all responsive to systemic steroids. All 5 patients are alive in complete remission without MRD.

## Discussion

This study demonstrates the feasibility of HLA-vaccine transplantations for refractory leukemia, extending beyond double-unit cord blood to haploidentical–cord blood or haploidentical–haploidentical HSCT. If validated in other cohorts, this approach may broaden accessibility, as suitable vaccine-effect double-cord units are not always available as illustrated in our cohort. Recent randomized studies have demonstrated favorable survival after haploidentical–cord blood HSCT without excessive toxicities (7, 8).

Despite refractoriness to contemporary therapies—including conventional HSCT, CAR-T cells, antibodies, and small molecules—all 5 patients achieved MRD-negative remission within 1 month of HLA-vaccine transplantation. The timing of leukemia clearance coincided with rapid LU rejection, supporting the hypothesis that LU provided an HLA-vaccine effect against leukemia cells sharing the mismatched HLA allotype. This hematopoietic cell-derived vaccine effect appeared relatively specific for hematologic malignancies, as clearance occurred in 2 of the 5 patients without clinical GVHD, consistent with prior reports (1, 2).

We purposefully selected graft pairs for vaccine effect regardless of WU dominance, preferring involvement of both MHC Class I and II when possible (**Table 2**) (2, 9). Mechanistically, in double-unit cord blood HSCT, prior studies showed WU-derived CD8^+^ T cells mediating graft-versus-graft (GvG) alloreactivity via mismatched class I against LU (10). Similarly, HLA class II–mismatched HSCT showed robust expansion of WU-derived CD4^+^ T cells against LU alleles in 29/31 recipients (11, 12). In both, WU-derived T cells were non-reactive against third-party cells, suggesting an HLA allotype–restricted GvG and GvL effect (10–12).

**Table 2:** An illustrative example of HLA-vaccine antigens in patient 1.

HLA	Patient		Haploidentical graft		UCB graft	
Haplotype	a	c	a	b	e	c
A	02:03	11:01	02:03	24:02	34:05	11:01
B	38:02	38:02	38:02	55:02	38:02	38:02
C	07:02	07:02	07:02	12:03	07:02	07:02
DRB1	16:02	15:02	16:02	13:12	15:02	15:02

*Potential HLA-vaccine antigens are underlined in red font. This cord blood unit was chosen based on 3 sets of criteria: [1] Fulfilment of the standard step-by-step CBU selection guide in the United States (Ref 13), including TNC >2.5x10^7^/kg, CD34 >1.5x10^5^/kg, HLA allele match at least 5/8, collection <15 years ago, RBC depleted unit, and accredited UCB bank, with units sorted by 8-allele HLA match (the best matched units available were 6/8 in GVHD direction in this case). [2] All the mismatched HLA alleles were preferably sited in the haplotype shared by the patient and the haploidentical graft (Haplotype a), involving both MHC Class I and II if possible; thus if the UCB was the WU, the HLA-A*02:03 and HLA-DRB1*16:02 in the haploideintical LU may serve as the vaccine antigens. [3] The matched HLA alleles were preferably sited in the haplotype that was not shared by the patient and the haploidentical graft (Haplotype c); thus if the haploidentical graft was the WU, the matched HLA alleles HLA-A*11:01 and HLA-DRB1*15:02 in the UCB that were absent in the haploidentical graft may serve as the vaccine antigens.

Although our findings support broader feasibility, limitations include the small cohort and short follow-up. While HLA-vaccine effect is the most plausible explanation for leukemia response, the multiagent non-TBI conditioning may have contributed despite refractory disease. Larger, longer-term studies are needed to confirm durability of remission. If validated, vaccine transplantation may represent a novel and powerful therapy for refractory leukemia lacking curative options.

## Data Availability

The original contributions presented in the study are included in the article/supplementary material. Further inquiries can be directed to the corresponding author.
